# Preparedness for early detection of oral cancer and oral potentially malignant disorders among Saudi dental students: a multicenter cross-sectional survey

**DOI:** 10.3389/fonc.2026.1914773

**Published:** 2026-09-30

**Authors:** Bassel Tarakji, Anas Alsalhani, Faisal Mehsen Alali, Nasser Raqe Alqhtani, Abdullah Saad Alqahtani, Abdullah bin nabhan, Abdullah Almansour, Abdulaziz Alrubayyi, Majed Kariri, Mohammed Said Alshahrei, Lina A. Alshahrani, Mohammed Alshammari, Rawda Omar Alghabban, Halah Thanoon, Deema Alshammery, Mohammad Zakaria Nassani, Faisal S. Alhedyan

**Affiliations:** 1 Department of Oral Maxillofacial Surgery and Diagnostic Sciences, College of Dentistry, Prince Sattam Bin Abdulaziz University, Al-Kharj, Saudi Arabia; 2 Department of Oral Pathology, Faculty of Dentistry, University of Aleppo, Aleppo, Syria; 3 Department of Dentistry, Vision Colleges, Riyadh, Saudi Arabia; 4 Department of Histology and Pathology, Faculty of Dentistry, University of Hama, Hama, Syria; 5 Department of Preventive Dental Sciences, College of Dentistry Prince Sattam Bin Abdulaziz University, Al-Kharj, Saudi Arabia; 6 College of Dentistry, Prince Sattam Bin Abdulaziz University, Al-Kharj, Saudi Arabia; 7 Faculty of Dentistry, King Saud, University, Riyadh, Saudi Arabia; 8 Department of Paediatric Dental Sciences, College of Dentistry, Prince Sattam Bin Abdulaziz University, Al-Kharj, Saudi Arabia; 9 Restorative Dentistry, Faculty of Dentistry, King Abdulaziz University, Jeddah, Saudi Arabia; 10 Department of Preventive Dentistry, College of Medicine and Dentistry, Riyadh Elm University, Riyadh, Saudi Arabia; 11 Department of Restorative and Prosthetic Dental Sciences, College of Dentistry, Dar Al Uloom University, Riyadh, Saudi Arabia; 12 Department of Removable Prosthodontics, Faculty of Dentistry, University of Aleppo, Aleppo, Syria

**Keywords:** dental students, diagnostic confidence, early detection, oral cancer, oral potentially malignant disorders, Saudi Arabia

## Abstract

**Background:**

Oral cancer remains a major global health challenge, with delayed diagnosis contributing to poor prognosis and reduced survival. Dental students play an important role in early detection; however, deficiencies in preparedness and diagnostic confidence have been reported. This study assessed oral cancer knowledge, oral potentially malignant disorder (OPMD) awareness, and diagnostic confidence among Saudi dental students.

**Methods:**

A quantitative cross-sectional online survey was conducted among 354 dental students and interns from governmental and private dental schools in Saudi Arabia. The questionnaire assessed oral cancer knowledge, OPMD knowledge, and self-reported diagnostic confidence related to screening and biopsy procedures. Participants were classified as meeting or not meeting a study-defined minimum operational threshold of ≥60% of the maximum possible score in each domain; this cutoff was not interpreted as a definition of “good” performance or clinical competence. Associations between participant characteristics and study outcomes were examined using chi-square tests and multivariable binary logistic regression analyses.

**Results:**

Overall, 81.9% of participants met the oral cancer knowledge threshold, 55.4% met the OPMD knowledge threshold, and 24.0% met the diagnostic confidence threshold. Most participants correctly identified major oral cancer risk factors, including tobacco smoking (97.2%), alcohol consumption (92.7%), and human papillomavirus infection (86.7%). However, clinically oriented response patterns highlighted areas for educational attention. 20.1% selected the prespecified specialist-referral response for management of a persistent suspicious lesion, while 35.6% recognized factors associated with increased malignant transformation risk in leukoplakia. Intern status remained independently associated with meeting the oral cancer knowledge and diagnostic confidence thresholds in multivariable analyses. The diagnostic-confidence outcome reflected self-perceived preparedness rather than observed screening, referral, or procedural competence.

**Conclusion:**

A high proportion of Saudi dental students met the study-defined oral cancer knowledge threshold; however, self-reported diagnostic confidence and preparedness for early detection remained considerably lower. Strengthening competency-based oral cancer education and clinical training may improve early detection preparedness and referral practices among future dental practitioners. Because the domains assessed different constructs and had different effective cutoffs, their threshold proportions should not be compared directly; the diagnostic-confidence findings represent perceived preparedness only.

## Introduction

Oral cancer is widely regarded as one of the most serious malignancies of the head and neck because of its substantial disease burden, the profound functional impairment it causes, and the poor survival rates associated with late-stage diagnosis. Although diagnostic and therapeutic modalities have advanced considerably, many patients still present at advanced stages, resulting in extensive tissue destruction, more complex treatment requirements, and diminished survival outcomes (1). As a result, strengthening early detection efforts has become a central priority in modern oral healthcare and cancer-prevention initiatives (2).

Dental practitioners serve as key frontline providers in oral cancer prevention, as routine dental appointments offer repeated opportunities for comprehensive oral mucosal screening and early detection of suspicious lesions A recent multicountry survey of 4, 197 adults across 13 Middle Eastern and North African countries found limited public awareness of oral cancer and showed that receiving oral cancer education from a dentist was strongly associated with higher knowledge across the assessed domains (3). Therefore, dental students must develop strong foundational knowledge and clinical competence during their undergraduate training to recognize potentially malignant changes, conduct systematic oral examinations, and make timely referrals for specialist evaluation or biopsy. Inadequate preparation at the undergraduate level may contribute to diagnostic delays and missed opportunities for early intervention (4).

A substantial proportion of oral cancers develop from pre-existing oral potentially malignant disorders (OPMDs) (5). Conditions such as leukoplakia, erythroplakia, oral submucous fibrosis, and oral lichen planus carry varying degrees of malignant transformation risk and therefore require meticulous clinical evaluation and long-term surveillance. Early identification of these disorders is critical, as timely intervention during the premalignant phase may support earlier recognition of malignant change and timely management (6).

Research conducted among dental students and dental professionals in different countries has demonstrated inconsistent levels of oral cancer awareness and diagnostic preparedness. Although awareness of major risk factors is often satisfactory (7, 8), deficiencies continue to be reported in practical areas such as biopsy-related procedures, management of suspicious lesions, referral pathways, and confidence in oral cancer screening practices (9). These findings indicate that acceptable theoretical understanding may not always correspond to adequate clinical readiness (10).

Educational progression appears to significantly influence oral cancer-related competence among dental students. Advanced academic levels and greater clinical exposure have been associated with improved diagnostic confidence and better recognition of oral mucosal abnormalities, suggesting that clinical training plays an important role in strengthening early detection capabilities (11).

Relevant studies from Türkiye provide regional educational context. A 2025 multicenter survey of 603 third-, fourth-, and fifth-year dental students from 10 faculties reported variation in oral cancer knowledge, self-perceived preparedness, and responses across both academic grades and faculties (12). Academic-level comparisons from Türkiye have also identified differences in recognition of oral cancer and potentially malignant/precancerous lesions: fifth-year students in Van more often recognized leukoplakia and erythroplakia as lesions with high carcinogenic potential than third-year students (13), and a recent first- versus fifth-year comparison found higher knowledge, screening awareness, and referral-related awareness among senior students (13). Because these studies used different questionnaires, populations, and academic-level definitions, their reported percentages are best used as contextual evidence rather than as directly comparable estimates.

In Saudi Arabia, evidence regarding oral cancer knowledge, awareness of OPMDs, and diagnostic confidence among dental students remains limited, particularly in relation to oral lesion management and biopsy-associated practices. Evaluating these aspects is important for identifying educational deficiencies and guiding curriculum development aimed at improving oral cancer screening competencies among future dental practitioners (14, 15).

Therefore, the present study aimed to assess oral cancer knowledge, knowledge of oral potentially malignant disorders (OPMDs), and diagnostic confidence among Saudi dental students. In addition, the study sought to examine demographic and academic factors associated with meeting the study-defined oral cancer knowledge, OPMD knowledge, and diagnostic confidence thresholds, with particular attention to the discrepancy between theoretical knowledge and clinical preparedness related to oral cancer screening and early detection. The contribution of this study is the integrated assessment of these three domains, including clinically oriented referral and biopsy items, within a common analytical framework across 20 reported institutional-affiliation categories.

## Materials and methods

### Study design and participants

A quantitative observational cross-sectional survey was carried out among dental students and interns enrolled in Saudi dental schools. The study was designed to evaluate participants’ understanding of oral cancer, knowledge of oral potentially malignant disorders (OPMDs), and confidence in performing oral cancer screening and managing suspicious oral lesions.

Participants were recruited using nonprobability convenience sampling. Data were collected from April 2024 through the end of December 2024. The target population comprised third-, fourth-, and fifth-year undergraduate dental students and dental interns at governmental and private dental schools in Saudi Arabia. A structured, self-administered questionnaire was hosted on Google Forms, and the survey link was distributed electronically through WhatsApp, Telegram, X, and student discussion groups to facilitate participation from dental institutions in different regions of the country. As invitations were disseminated through these channels without an individually tracked invitation list, the number of unique eligible students who received the invitation could not be established. Consequently, a conventional response rate could not be calculated. Participation was entirely voluntary, and responses were submitted anonymously after electronic consent was provided. To minimize duplicate submissions, the Google Forms ‘Limit to 1 response’ setting was enabled during data collection, restricting submissions to one per signed-in Google account. All questionnaire items were mandatory, and respondents were required to answer every item before the form could be submitted. The final analytical sample comprised 354 eligible respondents from 20 reported institutional-affiliation categories, including governmental and private institutions. Of these respondents, 251 were undergraduate dental students and 103 were interns. Institutional affiliation and university type were based on information reported by participants. Responses from the 30 participants involved in pilot testing were excluded from the final analysis.

Reporting of the study was guided by the Strengthening the Reporting of Observational Studies in Epidemiology (STROBE) guidelines (16) and the Checklist for Reporting Results of Internet E-Surveys (CHERRIES) (17). A completed CHERRIES checklist is provided as **Supplementary Material**. Ethical approval was obtained from the Institutional Review Board of Prince Sattam bin Abdulaziz University (Approval No. SCBR-373/2024). All procedures were performed in accordance with the Declaration of Helsinki. Participant anonymity was ensured, and all collected data were treated with strict confidentiality. This study involved human participants. Electronic informed consent was obtained from all participants before questionnaire completion, and participation was voluntary and anonymous.

### Questionnaire development, validation, and data collection

Data were gathered using a structured self-administered questionnaire distributed electronically through online platforms. The survey instrument was developed after reviewing previously published studies related to oral cancer awareness, oral potentially malignant disorders (OPMDs), and diagnostic preparedness among dental students and dental practitioners (18–21).

The questionnaire consisted of four sections:

1. Sociodemographic information.
2. Knowledge related to oral cancer.
3. Knowledge regarding oral potentially malignant disorders (OPMDs).
4. Diagnostic confidence and preparedness for oral cancer screening and biopsy-related procedures.

The questionnaire items were presented in multiple-choice format with predefined response options. No adaptive questioning or branching logic was used. All participants were presented with the same questionnaire items. The survey evaluated participants’ understanding of oral cancer risk factors, clinical manifestations, lesion monitoring, biopsy procedures, identification of OPMDs, malignant transformation potential, and self-perceived confidence in oral cancer examination and early detection practices.

The complete questionnaire, original response options, designated scoring key, and supporting clinical references are provided in the **Supplementary Questionnaire**. For the item addressing management of a persistent suspicious lesion, “Consultation to a Specialist” was the prespecified scoring key. Because this was an educational survey of third-, fourth-, and fifth-year dental students and dental interns, the item was intended to assess referral-related decision-making and recognition of the need for escalation to specialist evaluation rather than to define the full range of management actions available to an appropriately trained clinician. Current evidence-based guidance supports either immediate biopsy by an appropriately trained clinician or immediate referral to an appropriate specialist when an oral mucosal lesion is clinically suspicious (22). For the follow-up item, 3 weeks was the prespecified scoring key, reflecting NICE guidance that unexplained oral ulceration persisting for more than 3 weeks warrants referral on a suspected-cancer pathway. In this student/intern educational context, the item is interpreted as recognition of a persistence threshold rather than as a recommendation to observe an already clinically suspicious lesion for 3 weeks; such lesions require prompt biopsy or specialist referral (23). For the malignant-transformation item, erosive oral lichen planus was designated as the lowest-risk option among the listed choices; this comparative classification is consistent with the relatively low absolute malignant-transformation rate reported for oral lichen planus compared with several higher-risk OPMDs, while erosive/red forms still warrant continued surveillance (7, 44).

Content validity and face validity were assessed by three experts in the field of oral medicine and oral pathology to ensure the relevance, clarity, and appropriateness of the questionnaire items. A pilot study involving 30 participants was subsequently conducted to evaluate questionnaire clarity, comprehensibility, and feasibility. Based on participant feedback obtained during the pilot phase, minor modifications were made to improve the wording and structure of several items. Responses collected during the pilot study were excluded from the final statistical analysis (17).

### Scoring and operational definitions

Each response matching the prespecified scoring key in the oral cancer and OPMD knowledge domains was assigned one point. Diagnostic confidence items were dichotomized. For the three Poor/Fair/Good self-rating items, the response option “Good” was coded as a favorable item-level response; for the oral-biopsy knowledge self-rating item, “Yes” was coded as favorable. These item-level favorable responses were distinct from the aggregate domain classification and did not mean that participants meeting the ≥60% study-defined threshold had “good” diagnostic competence.

Bloom’s taxonomy provides a framework for educational objectives (24); it was not used to validate a numerical cutoff for the present questionnaire. A threshold of ≥60% was applied as a pragmatic, study-defined operational criterion for descriptive comparisons and binary logistic regression analyses. This choice has precedent in healthcare questionnaire research: Raab et al. (25) classified knowledge using a threshold of at least three of five questions answered correctly or sufficiently. In Saudi dental hygiene students, Mulla (26) categorized scores <60% as poor, 60%–80% as average, and >80% as good. These studies provide precedent for using a 60% boundary, rather than validation of the present domain classifications. Accordingly, aggregate outcomes are described as meeting or not meeting the study-defined threshold and are not interpreted as evidence of clinical competence.

Because the domain scores were based on whole items, the nominal ≥60% rule did not produce an exact 60% cutoff in any domain. Participants meeting the study-defined threshold scored ≥8/12 for oral cancer knowledge (66.7%) and ≥9/14 for OPMD knowledge (64.3%). For diagnostic confidence, the same rule required ≥3/4 favorable self-reported responses (75.0%). Thus, the effective operational cutoffs ranged from 64.3% to 75.0%. Because diagnostic confidence was self-reported rather than performance-based, this classification represents perceived preparedness and was not validated against an external measure of clinical competence.

### Reliability analysis

Internal consistency reliability was evaluated in the main study sample using Cronbach’s alpha coefficient in IBM SPSS Statistics version 25. Reliability testing was performed on item-level responses within each questionnaire domain, with reverse coding applied where appropriate prior to analysis. The questionnaire demonstrated acceptable internal consistency reliability. Domain-specific Cronbach’s alpha coefficients were 0.72 for oral cancer knowledge, 0.74 for knowledge of oral potentially malignant disorders (OPMDs), and 0.71 for diagnostic confidence. Cronbach’s alpha values of ≥0.70 were considered indicative of acceptable reliability for the study instrument (27).

### Sample size calculation

The theoretical sample size requirement for estimating a single proportion was assessed using the single-proportion formula implemented in OpenEpi software (Open Source Epidemiologic Statistics for Public Health, version 3.01) (28). The value of 50% used in the calculation did not represent the prevalence of oral cancer or OPMDs. Rather, because no sufficiently reliable prior estimate was available for the proportion of Saudi dental students expected to meet the study outcomes across the assessed domains, an anticipated proportion of 50% (p = 0.50) was selected as a conservative assumption. This value maximizes p(1 - p) and therefore yields the largest required sample size for a fixed confidence level and margin of error. Using n = Z²p(1 - p)/d², with a 95% confidence level (Z = 1.96), p = 0.50, and an absolute precision of 5%, the calculation yielded 384.16, rounded upward to 385 participants. The final analytical sample comprised 354 dental students and interns. Under the same simple-random-sampling assumptions, this sample corresponds to a nominal margin of error of approximately ±5.21 percentage points. This precision estimate represents a theoretical benchmark given the study’s nonprobability convenience sampling approach.

A supplementary assessment evaluated the available sample relative to the complexity of the multivariable logistic regression models. Each model included three binary predictors: gender, study level, and university type, corresponding to three predictor parameters excluding the intercept. The smaller outcome categories contained 64 participants for oral cancer knowledge, 158 for OPMD knowledge, and 85 for diagnostic confidence. Dividing these counts by three yielded ratios of 21.3, 52.7, and 28.3 observations per predictor parameter, respectively. These ratios exceeded the conventional benchmark of 10 events per predictor parameter (29), supporting the limited complexity of the fitted models. Nevertheless, this assessment does not establish statistical power for individual adjusted associations or guarantee model adequacy (30).

### Statistical analysis

Data analysis was performed using IBM SPSS Statistics version 25.0 (IBM Corp., Armonk, NY, USA). Descriptive statistics were applied to summarize participants’ demographic characteristics and questionnaire responses. Categorical variables were reported as frequencies and percentages.

Item scores were summed within each domain, and participants were classified as meeting or not meeting a study-defined minimum threshold of ≥60% of the maximum possible domain score. This boundary was used as an operational classification criterion, informed by previous questionnaire research (22, 23), and was not considered a validated standard of good performance. Because whole-item scoring was used, the corresponding effective thresholds were ≥8/12 (66.7%) for oral cancer knowledge, ≥9/14 (64.3%) for OPMD knowledge, and ≥3/4 (75.0%) favorable self-reported responses for diagnostic confidence. As a complementary descriptive measure, mean raw domain scores were also summarized from the item-level responses and expressed as both raw scores and percentages of each domain maximum; for diagnostic confidence, this represented the mean number of favorable self-reported responses rather than an objective performance score.

Associations between participant characteristics and meeting the oral cancer knowledge, OPMD knowledge, and diagnostic confidence thresholds were assessed using the chi-square (χ²) test with Yates’s continuity correction for 2 × 2 tables (**Table 1**).

**Table 1:** Associations between dental students’ characteristics and meeting the study-defined oral cancer knowledge, OPMD knowledge, and diagnostic confidence thresholds (n = 354).

Characteristics	Met oral cancer knowledge threshold		Met OPMD knowledge threshold		Met diagnostic confidence threshold	
	n (%)	*p*	n (%)	*p*	n (%)	*p*
Gender						
Female	160 (82.1)	1.000	109 (55.9)	0.909	37 (19.0)	0.020*
Male	130 (81.8)		87 (54.7)		48 (30.2)	
Study level						
Intern	93 (90.3)	0.014*	57 (55.3)	1.000	38 (36.9)	<0.001*
Undergraduate	197 (78.5)		139 (55.4)		47 (18.7)	
University						
Governmental	245 (85.4)	0.001*	156 (54.4)	0.512	65 (22.6)	0.278
Private	45 (67.2)		40 (59.7)		20 (29.9)	

OPMDs, oral potentially malignant disorders. Outcomes were defined as meeting the study-defined minimum threshold of at least 60% of the maximum possible domain score, corresponding to ≥8/12 (66.7%) for oral cancer knowledge, ≥9/14 (64.3%) for OPMD knowledge, and ≥3/4 (75.0%) favorable self-reported responses for diagnostic confidence. The ≥60% rule was used as an operational threshold and should not be interpreted as a definition of “good” knowledge or clinical competence. P values were calculated using the chi-square test with Yates’s continuity correction for 2 × 2 tables. P < 0.05 was considered statistically significant. *Denotes a statistically significant association.

Subsequently, multivariable binary logistic regression analyses were performed to identify independent predictors of meeting each study-defined threshold. The independent variables included gender, study level, and university type. Adjusted odds ratios (ORs) with corresponding 95% confidence intervals (CIs) were calculated (**Table 2**).

**Table 2:** Multivariable logistic regression analysis of factors associated with meeting the study-defined oral cancer knowledge, oral potentially malignant disorder (OPMD) knowledge, and diagnostic confidence thresholds among dental students (n = 354).

	Met oral cancer knowledge threshold		Met OPMD knowledge threshold		Met diagnostic confidence threshold	
Characteristics	OR (95% CI)	*p*	OR (95% CI)	*p*	OR (95% CI)	*p*
Gender						
Female	Reference		Reference		Reference	
Male	1.02 (0.58–1.79)	0.944	1.05 (0.68–1.63)	0.815	1.82 (1.09–3.05)	0.022*
Study level						
Intern	Reference		Reference		Reference	
Undergraduate	0.39 (0.20–0.77)	0.007*	0.99 (0.61–1.59)	0.954	0.39 (0.23–0.67)	<0.001*
University type						
Governmental	Reference		Reference		Reference	
Private	0.35 (0.19–0.66)	0.001*	1.25 (0.74–2.14)	0.408	1.45 (0.80–2.64)	0.221

OR, odds ratio; CI, confidence interval; OPMD, oral potentially malignant disorder.

Odds ratios (ORs) and 95% confidence intervals (CIs) were estimated using multivariable binary logistic regression models. Each outcome denotes meeting the study-defined minimum threshold (≥8/12 [66.7%] for oral cancer knowledge, ≥9/14 [64.3%] for OPMD knowledge, and ≥3/4 [75.0%] favorable self-reported responses for diagnostic confidence), not a “good” performance or clinical-competence category. Asterisk denotes statistical significance (p < 0.05).

Multicollinearity among gender, study level, and university type was assessed using variance inflation factors (VIFs), interpreted as measures of dependence among the predictor variables (31). The Hosmer–Lemeshow statistics from an earlier reanalysis were not used as goodness-of-fit evidence for **Table 2** because they were obtained from different fitted models.

All statistical tests were two-tailed, and statistical significance was set at p < 0.05.

## Results

### Participant characteristics

A total of 354 Saudi dental students and interns were included in the final analysis. Female participants constituted 55.1% (n = 195) of the study population, whereas males represented 44.9% (n = 159). Undergraduate dental students accounted for 70.9% (n = 251) of participants, while interns represented 29.1% (n = 103). Most participants were enrolled in governmental universities (81.1%, n = 287), whereas 18.9% (n = 67) attended private universities (**Table 3**).

**Table 3:** Characteristics of dental students included in the study (n = 354).

Characteristics	n	%
Gender		
Female	195	55.1%
Male	159	44.9%
Study level		
Intern	103	29.1%
Undergraduate	251	70.9%
University		
Governmental	287	81.1%
Private	67	18.9%

Respondents represented 20 reported institutional-affiliation categories. Counts for each affiliation and administrative region are provided in **Supplementary Tables S1, S2**; Riyadh contributed 300 respondents (84.7%), Makkah 27 (7.6%), and the other represented regions contributed 27 respondents (7.6%), with the merged Vision/Al-Farabi affiliation classified in Riyadh.

### Oral cancer knowledge, OPMD knowledge, and diagnostic confidence

Tobacco smoking was correctly identified as an oral cancer risk factor by 97.2% of participants, and alcohol consumption by 92.7%. The lateral tongue was identified as the most common oral cancer site by 91.8%. In the OPMD domain, 95.2% recognized the association between betel quid and oral submucous fibrosis, and 90.4% recognized that histopathological examination is required to confirm dysplasia in leukoplakia (**Table 4**).

**Table 4:** Number and percentage of dental students demonstrating correct knowledge regarding oral cancer and oral potentially malignant disorders (OPMDs), and favorable diagnostic-confidence responses (n = 354).

Domains and items	Correct/favorable responses	
	Number	Percent
Oral cancer knowledge		
Ultraviolet exposure as a risk factor for oral cancer (Yes)	302	85.3%
Tobacco smoking as a risk factor for oral cancer (Yes)	344	97.2%
Alcohol consumption as a risk factor for oral cancer (Yes)	328	92.7%
HPV infection as a risk factor for oral cancer (Yes)	307	86.7%
Age >40 years as a risk factor for oral cancer (Yes)	247	69.8%
Most common site of oral cancer (Lateral tongue)	325	91.8%
Most common oral cancer (Squamous cell carcinoma)	200	56.5%
Clinical feature of OSCC (Red-ulcerative lesion)	307	86.7%
Most important microscopic feature (Dysplasia)	274	77.4%
Management of persistent suspicious lesion (Specialist referral)	71	20.1%
Follow-up period for suspicious lesion (3 weeks)	198	55.9%
Biopsy specimen storage medium (Formalin)	283	79.9%
Met oral cancer knowledge threshold	290	81.9%
Knowledge of OPMD		
Recognition of non-precancerous lesion (Aphthous ulcer)	259	73.2%
Recognition of leukoplakia as OPMD	262	74.0%
Recognition of erythroplakia as OPMD	239	67.5%
Recognition of oral submucous fibrosis as OPMD	287	81.1%
Recognition of oral lichen planus as OPMD	218	61.6%
High-risk leukoplakia subtype (Proliferative verrucous leukoplakia)	297	83.9%
High malignant transformation lesion (Erythroplakia)	244	68.9%
Low malignant transformation lesion (Erosive lichen planus)	166	46.9%
Risk factor increasing leukoplakia malignant transformation	126	35.6%
Malignant transformation more common in females	135	38.1%
Ventrolateral tongue as high-risk site	242	68.4%
Betel quid associated with oral submucous fibrosis	337	95.2%
Leukoplakia requires histopathology for dysplasia confirmation	320	90.4%
Nonsmoker leukoplakia may have higher malignant potential	259	73.2%
Met OPMD knowledge threshold	196	55.4%
Diagnostic Confidence		
Self-rated oral cancer knowledge (Good)	176	49.7%
Self-rated clinical detection skills (Good)	151	42.7%
Self-rated preparedness for early detection (Good)	136	38.4%
Knowledge regarding oral biopsy techniques (Good)	117	33.1%
Met diagnostic confidence threshold	85	24.0%

Participants were classified as meeting a study-defined minimum threshold of at least 60% of the maximum possible domain score. This was an operational minimum, not a definition of “good” knowledge or clinical competence. Because scores were based on whole items, the effective thresholds were ≥8/12 (66.7%) for oral cancer knowledge, ≥9/14 (64.3%) for OPMD knowledge, and ≥3/4 favorable self-reported responses (75.0%) for diagnostic confidence. The word “Good” shown for individual diagnostic-confidence items reflects the questionnaire response option only and is distinct from the aggregate threshold classification. HPV, human papillomavirus; OSCC, oral squamous cell carcinoma; OPMDs, oral potentially malignant disorders.

Regarding oral potentially malignant disorders (OPMDs), 83.9% correctly identified proliferative verrucous leukoplakia as a high-risk subtype, whereas 81.1% recognized oral submucous fibrosis as an OPMD (**Table 4**). Several clinically oriented response patterns highlighted areas for educational attention. 20.1% selected the prespecified specialist-referral response for management of a persistent suspicious lesion, while 35.6% correctly recognized factors associated with increased malignant transformation risk of leukoplakia. In addition, only 38.1% correctly identified female gender as being associated with a higher malignant transformation risk in leukoplakia lesions.

Diagnostic confidence levels were considerably lower than the knowledge outcomes. Only 49.7% of participants reported “good” self-rated oral cancer knowledge, 42.7% reported “good” self-rated clinical detection skills, 38.4% reported feeling prepared for early oral cancer detection, and 33.1% reported “good” knowledge of oral biopsy techniques (**Table 4**). These responses measured self-perception and were not observed assessments of screening, referral, biopsy, or diagnostic competence.

### Proportions meeting the study-defined thresholds

Using the study-defined thresholds, 81.9% (n = 290) of participants met the oral cancer knowledge threshold, 55.4% (n = 196) met the OPMD knowledge threshold, and 24.0% (n = 85) met the diagnostic confidence threshold (**Table 4**). As a complementary continuous description, the mean raw domain scores were 9.00/12 (75.0% of the maximum) for oral cancer knowledge, 9.58/14 (68.4%) for OPMD knowledge, and 1.64/4 favorable self-reported responses (41.0%) for diagnostic confidence. These mean scores are descriptive within each domain and do not make the three constructs directly comparable.

### Associations with study-defined thresholds

No statistically significant associations were observed between gender and meeting the oral cancer knowledge threshold (p = 1.000) or the OPMD knowledge threshold (p = 0.909). However, males were more likely than females to meet the diagnostic confidence threshold (30.2% vs. 19.0%, p = 0.020).

Academic level was significantly associated with some outcomes. Interns had higher proportions meeting the oral cancer knowledge threshold (90.3% vs. 78.5%, p = 0.014) and the diagnostic confidence threshold (36.9% vs. 18.7%, p < 0.001) than undergraduate students. No statistically significant difference was observed between interns and undergraduates in meeting the OPMD knowledge threshold (55.3% vs. 55.4%, p = 1.000). These differences according to academic level are illustrated in **Figure 1**.

**Figure 1:**
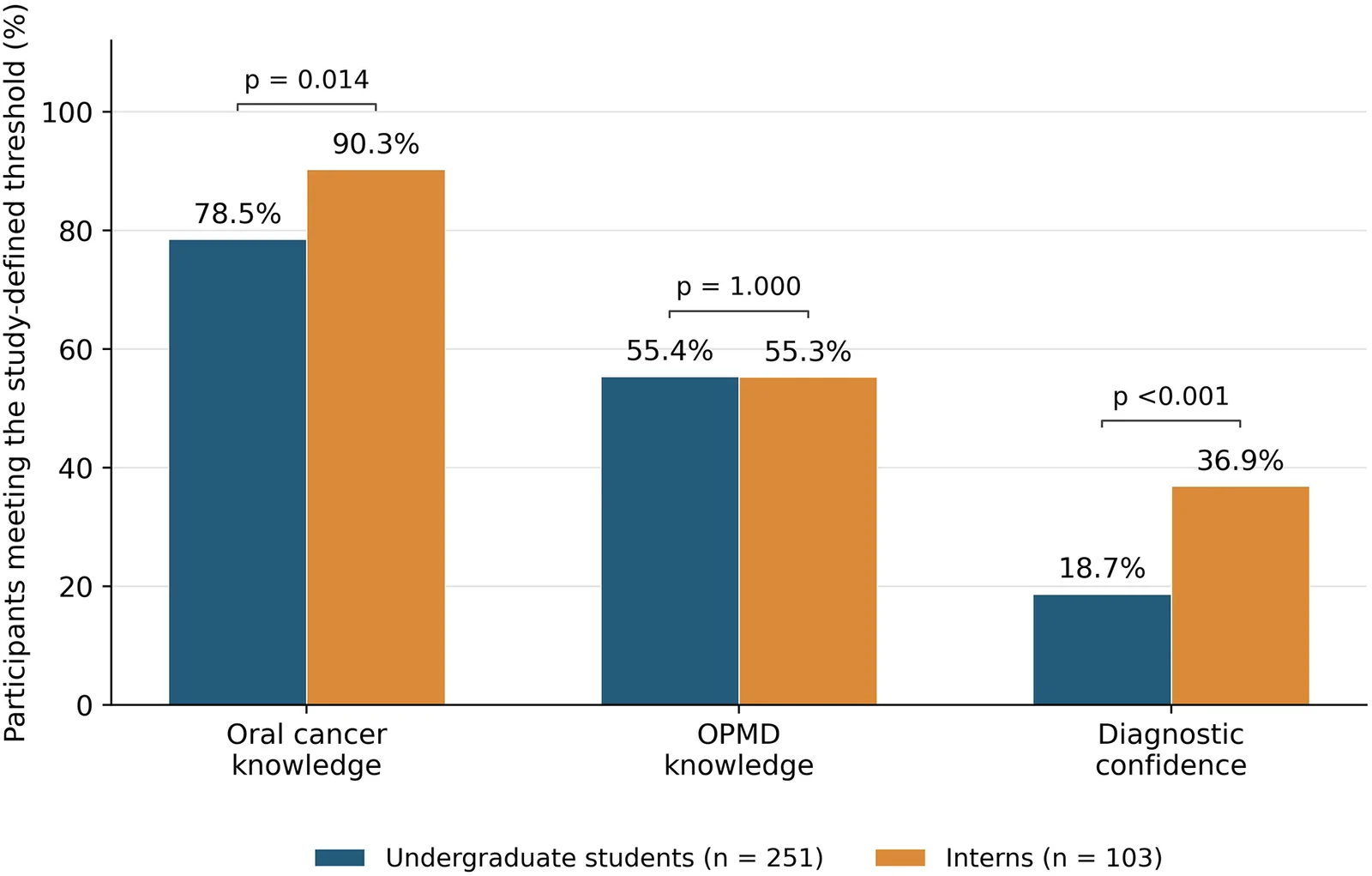
Proportions of participants meeting the study-defined oral cancer knowledge, OPMD knowledge, and diagnostic confidence thresholds according to academic level. Bars represent the percentages of undergraduate students and interns meeting each study-defined threshold. P values were derived from chi-square tests with Yates's continuity correction, as reported in **Table 3**. Thresholds were ≥8/12 (66.7%) for oral cancer knowledge, ≥9/14 (64.3%) for OPMD knowledge, and ≥3/4 favorable self-reported responses (75.0%) for diagnostic confidence.

A statistically significant association was found between university type and meeting the oral cancer knowledge threshold (85.4% for governmental versus 67.2% for private universities, p = 0.001). No statistically significant associations were found between university type and meeting the OPMD knowledge threshold (p = 0.512) or the diagnostic confidence threshold (p = 0.278). The corresponding differences by university type are shown in **Figure 2**.

**Figure 2:**
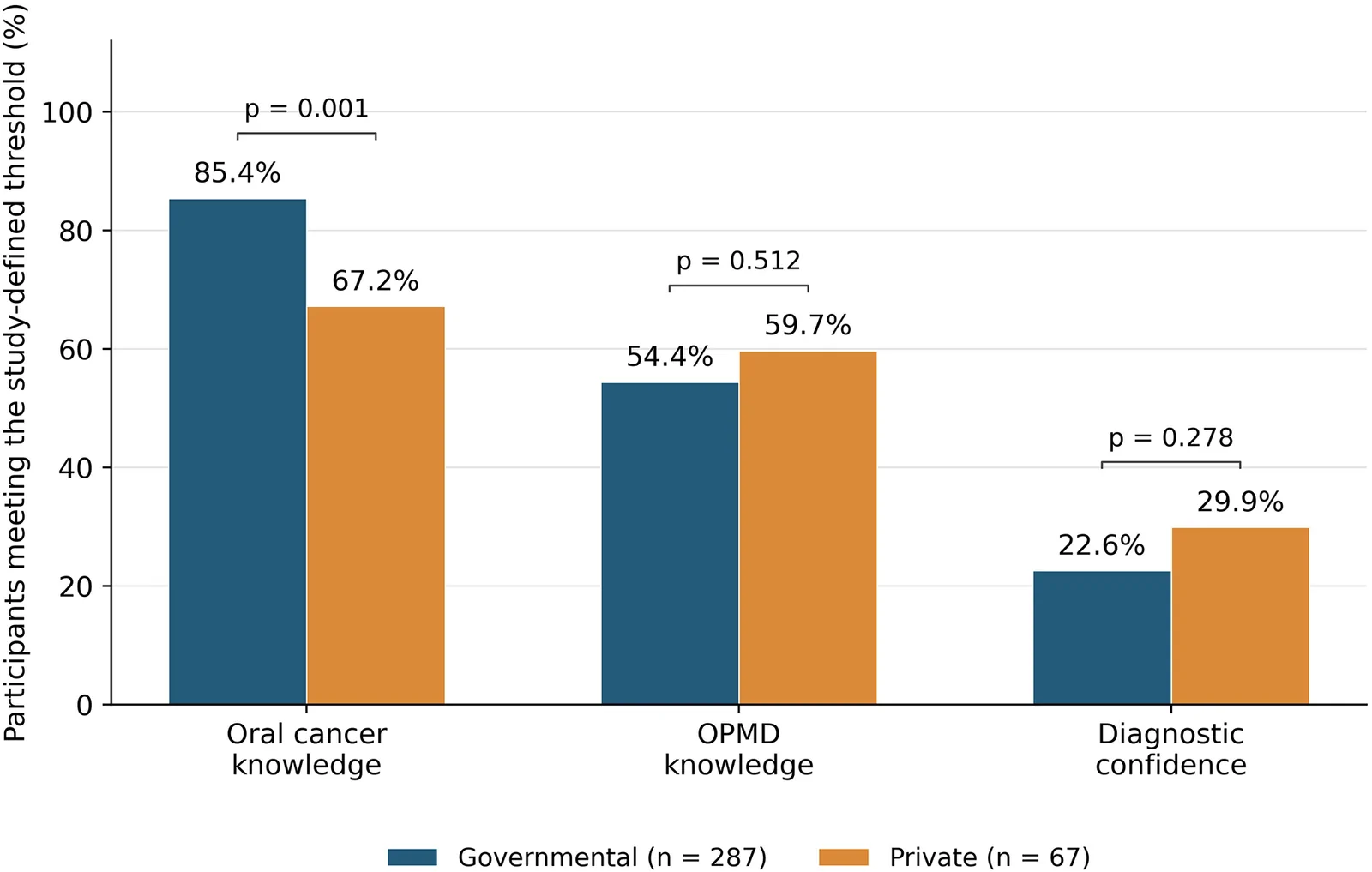
Proportions of participants meeting the study-defined oral cancer knowledge, OPMD knowledge, and diagnostic confidence thresholds according to university type. Bars represent the percentages of participants from governmental and private universities meeting each study-defined threshold. P values were derived from chi-square tests with Yates's continuity correction, as reported in **Table 3**. Thresholds were ≥8/12 (66.7%) for oral cancer knowledge, ≥9/14 (64.3%) for OPMD knowledge, and ≥3/4 favorable self-reported responses (75.0%) for diagnostic confidence.

### Multivariable logistic regression analysis

Multivariable logistic regression analysis, using females, interns, and students from governmental universities as the reference groups, showed that undergraduate students were significantly less likely to meet the oral cancer knowledge threshold (OR = 0.39, 95% CI: 0.20–0.77, p = 0.007) and the diagnostic confidence threshold (OR = 0.39, 95% CI: 0.23–0.67, p < 0.001) than interns. Similarly, students attending private universities were significantly less likely to meet the oral cancer knowledge threshold than those attending governmental universities (OR = 0.35, 95% CI: 0.19–0.66, p = 0.001). In contrast, male students were significantly more likely to meet the diagnostic confidence threshold than female students (OR = 1.82, 95% CI: 1.09–3.05, p = 0.022).

Study level, university type, and gender were not significantly associated with meeting the OPMD knowledge threshold in the adjusted regression model (**Table 2**).

VIF values ranged from 1.005 to 1.046, with a maximum of 1.046, indicating no evidence of problematic multicollinearity among gender, study level, and university type. VIF assesses dependence among predictors and does not establish model calibration.

## Discussion

### Strengths and methodological contributions of the present study

This multicenter study assessed oral cancer knowledge, knowledge of oral potentially malignant disorders (OPMDs), and self-reported diagnostic confidence within a single analytical framework. Participants were drawn from governmental and private dental institutions across Saudi Arabia, broadening institutional representation without implying population representativeness because recruitment was based on convenience sampling and institutional participation was unequal (19, 20). The questionnaire was informed by previous literature, reviewed by experts for face and content validity, pilot tested, and demonstrated acceptable internal consistency, supporting interpretation of the measured domains (17, 32, 33).

Multivariable logistic regression was used to examine independent associations with the study-defined thresholds. Academic level and university type were independently associated with oral cancer knowledge, whereas academic level and gender were associated with diagnostic confidence. No significant adjusted associations were identified for OPMD knowledge (9, 11).

### Knowledge, OPMD awareness, and diagnostic confidence

The three measured domains showed distinct patterns: 81.9% of participants met the oral cancer knowledge threshold, 55.4% met the OPMD knowledge threshold, and 24.0% met the diagnostic confidence threshold (**Table 4**). These proportions are not directly comparable because the domains assess different constructs and have different effective cutoffs. Importantly, diagnostic confidence reflects self-perceived preparedness rather than objectively demonstrated clinical performance and should not be interpreted as evidence of diagnostic accuracy or clinical competence. The continuous score summaries were therefore used only as within-domain descriptive complements to the dichotomized outcomes and not as evidence of equivalent performance across domains.

Although knowledge of major oral cancer risk factors was generally high, clinically oriented areas for educational attention remained. 20.1% selected the prespecified specialist-referral response for a persistent suspicious lesion, 35.6% identified factors associated with increased malignant transformation risk in leukoplakia, and 33.1% answered “Yes” to having sufficient knowledge about oral biopsy techniques (**Table 4**). Referral and biopsy-related preparedness has likewise been identified as an educational concern internationally (9, 10), underscoring the value of training in clinical decision-making.

These clinically oriented items should be interpreted within the educational context of a survey of dental students and interns and according to their prespecified scoring keys: specialist referral is a guideline-concordant response for a clinically suspicious lesion, although immediate biopsy by an appropriately trained clinician is also an acceptable pathway (22); the 3-week item represents a referral threshold for unexplained oral ulceration rather than a recommended delay for an already suspicious lesion (23); and erosive oral lichen planus was scored as the lowest-risk option among the disorders listed in that question, rather than as a lesion with negligible malignant potential (7, 44).

OPMD knowledge was lower than oral cancer knowledge. Gaps in understanding malignant transformation are clinically important because recognition and risk assessment of OPMDs are central to prevention and early intervention (5, 6). Comparable OPMD-related knowledge gaps have been reported among dental students in other settings (18, 20).

The Türkiye literature provides a useful regional parallel for these educational gaps. In a study comparing third- and fifth-year dental students, recognition of leukoplakia and erythroplakia as lesions with high carcinogenic potential was substantially more frequent among fifth-year students (13). This supports the relevance of academic progression to awareness of potentially malignant lesions, but the different instruments and sampled populations preclude direct comparison of percentages with the present study.

### Factors associated with study outcomes

Academic progression was related to both oral cancer knowledge and diagnostic confidence. Interns were more likely than undergraduate students to meet the corresponding thresholds, and these associations remained significant after adjustment (**Tables 1**, **2**). Greater clinical exposure during internship may contribute to these differences, consistent with studies linking advanced training and practical experience to improved oral cancer knowledge and confidence (7, 11, 34). However, because the study was cross-sectional, these associations should not be interpreted as evidence that academic progression or clinical exposure caused the observed differences.

This pattern is also consistent with recent evidence from Türkiye. The multicenter study by Yüksel et al. included students from 10 dental faculties and found significant variation across both academic grades and faculties (45), while Yildirim et al. reported higher oral cancer knowledge, screening awareness, and appropriate referral behavior among fifth-year than first-year students (47). These findings support examining training stage and institutional context, although differences in questionnaire content, sampling, and outcome definitions mean that the numerical results should not be treated as directly comparable.

Students from governmental universities were more likely than those from private universities to meet the oral cancer knowledge threshold, whereas university type was not associated with OPMD knowledge or diagnostic confidence after adjustment. Gender was not associated with either knowledge outcome; however, male participants were more likely to meet the self-reported diagnostic confidence threshold. These associations may also be influenced by unmeasured educational or participant-level factors and by the voluntary convenience-based recruitment strategy. As confidence was self-reported, the observed gender difference should be interpreted as a difference in perceived preparedness rather than objective clinical ability (10, 11).

### Educational, clinical, and public health implications

The findings support greater emphasis on competency-based oral cancer education, including structured oral mucosal examination, referral decision-making, assessment of malignant transformation risk, and biopsy-related training. Supervised clinical exposure, simulation-based learning, and practical workshops may help translate theoretical knowledge into greater clinical preparedness (35–37).

These priorities are clinically relevant because dentists are frontline providers for oral mucosal examination and early referral. Strengthening practical training during undergraduate education and internship may improve future screening and referral practices and help reduce diagnostic delay (38–43).

## Limitations

Several limitations should be considered when interpreting the present findings. First, the cross-sectional design provides a snapshot of associations at a single point in time and does not establish temporality or causality. Accordingly, associations between academic level, university type, gender, and the study outcomes should not be interpreted as causal effects. Second, the study relied on self-reported questionnaire responses; therefore, the findings may be affected by recall, social desirability, and self-assessment biases.

Recruitment was voluntary and conducted online through nonprobability convenience channels, which may have introduced self-selection and selection bias because students who encountered the survey link and chose to participate may differ systematically from nonparticipants in their interest, knowledge, or perceived preparedness. Because invitations were not individually tracked, the number of unique eligible students reached and a conventional response rate could not be determined, limiting assessment of nonresponse bias. Although respondents represented 20 reported institutional-affiliation categories, unequal participation across institutions and the convenience-based sampling strategy limit the generalizability of the findings to all dental students and interns in Saudi Arabia. The achieved sample of 354 participants was below the theoretical requirement of 385 for 5% absolute precision. Although the outcome counts supported the specified regression model complexity, this assessment did not establish power to detect modest adjusted associations. Nonsignificant findings should therefore be interpreted cautiously and with reference to their confidence intervals.

Diagnostic confidence was assessed using self-reported responses. The study also did not evaluate potentially influential educational factors, such as previous oral medicine training, clinical exposure to oral mucosal lesions, or participation in oral cancer workshops. Accordingly, associations involving this domain describe differences in perceived confidence/preparedness and cannot establish differences in observed screening or referral competence.

Because no objective performance-based assessment, such as a direct clinical examination, standardized case-based evaluation, or observed procedural task, was undertaken, the study cannot determine participants’ actual diagnostic accuracy or clinical competence. Diagnostic confidence should therefore be interpreted strictly as perceived preparedness and not as a surrogate measure of clinical performance.

The reported proportions meeting the knowledge and diagnostic confidence thresholds depend on the operational cutoffs used. The ≥60% rule was treated as a minimum operational threshold rather than a definition of good performance; whole-item scoring resulted in effective cutoffs of 64.3%–75.0% across the three domains. Using a more stringent criterion, such as ≥80%, could reduce the proportions meeting the thresholds and could alter estimated associations. Differences in domain content and effective cutoffs also limit direct comparisons of these proportions. Accordingly, these classifications should be interpreted as questionnaire-based operational outcomes and not as evidence of high-level knowledge or objective clinical competence. The regression findings should also be interpreted without an accompanying model-specific calibration assessment.

Nevertheless, despite these limitations, the present study provides important insight from this multicenter sample into oral cancer knowledge, OPMD awareness, and diagnostic confidence among Saudi dental students and identifies important educational gaps that may help guide future curriculum development and competency-based oral cancer training initiatives.

Future research should evaluate the effectiveness of targeted educational interventions, competency-based oral cancer training programs, and simulation-based clinical workshops (44) in improving diagnostic confidence and practical oral cancer screening skills among dental students. Longitudinal multicenter studies assessing students before and after structured educational interventions may provide further insight into the most effective strategies for strengthening oral cancer preparedness within undergraduate dental curricula.

In addition, future investigations should incorporate objective clinical competency assessments, including direct clinical examinations, case-based evaluations, and biopsy-related practical training, rather than relying solely on self-reported confidence measures. Further research may also explore the influence of curriculum structure, clinical exposure, oral medicine rotation experience, and participation in oral cancer awareness programs on diagnostic preparedness and early detection competency among dental students and young dental practitioners.

Finally, broader regional and international comparative studies may help identify educational variations and best practices for improving oral cancer education and early detection preparedness within dental schools.

## Conclusion

The present study found that a high proportion of Saudi dental students met the study-defined oral cancer knowledge threshold, whereas a smaller proportion met the OPMD knowledge threshold. Self-reported diagnostic confidence was lower, and clinically oriented areas for educational attention remained, particularly in responses to suspicious-lesion management items, malignant transformation risk assessment, and biopsy-related procedures. Intern status was associated with meeting the oral cancer knowledge and diagnostic confidence thresholds. However, given the cross-sectional design, voluntary online convenience recruitment, and reliance on self-reported measures, these findings should be interpreted as descriptive and associational rather than causal, and diagnostic confidence should not be regarded as equivalent to objectively demonstrated clinical competence. The diagnostic-confidence findings therefore describe self-perceived preparedness rather than observed competence, and the threshold proportions across the three domains should not be interpreted as directly comparable because their effective cutoffs differed.

These findings emphasize the need to strengthen competency-based oral cancer education within undergraduate dental curricula in Saudi Arabia through greater incorporation of supervised oral examination training, oral mucosal lesion assessment, biopsy-related education, and simulation-based clinical learning. Improving practical preparedness among future dental practitioners may contribute to earlier oral cancer detection, more effective referral practices, and improved public health outcomes.

## Data Availability

The raw data supporting the conclusions of this article will be made available by the authors, without undue reservation.
