# Supplementary material for: Preparedness for early detection of oral cancer and oral potentially malignant disorders among Saudi dental students: a multicenter cross-sectional survey
Source: Front Oncol. 2026 Sep 30;16:1914773. doi: 10.3389/fonc.2026.1914773 (PMC13661908; doi:10.3389/fonc.2026.1914773)
Supplement: Supplementary file 1 [file SupplementaryFile1.docx]

**Supplementary Tables**

**Supplementary Table S1. Respondents by reported institutional affiliation (n = 354)**

| **Reported institutional affiliation** | **n** | **%** | **Administrative region** |
| --- | --- | --- | --- |
| King Saud University | 98 | 27.7 | Riyadh |
| King Saud bin Abdulaziz University for Health Sciences | 64 | 18.1 | Riyadh |
| Prince Sattam bin Abdulaziz University | 38 | 10.7 | Riyadh |
| Vision Colleges (formerly Al-Farabi Colleges) | 35 | 9.9 | Riyadh |
| Majmaah University | 26 | 7.3 | Riyadh |
| Riyadh Elm University | 23 | 6.5 | Riyadh |
| Umm Al-Qura University | 19 | 5.4 | Makkah |
| Princess Nourah bint Abdulrahman University | 13 | 3.7 | Riyadh |
| University of Hail | 8 | 2.3 | Ha'il |
| Jazan University | 6 | 1.7 | Jazan |
| King Abdulaziz University | 5 | 1.4 | Makkah |
| King Khalid University | 5 | 1.4 | Asir |
| Riyadh Colleges of Dentistry and Pharmacy | 3 | 0.8 | Riyadh |
| Taibah University | 3 | 0.8 | Madinah |
| King Faisal University | 2 | 0.6 | Eastern Province |
| Taif University | 2 | 0.6 | Makkah |
| Al Baha University | 1 | 0.3 | Al Baha |
| Ibn Sina National College for Medical Studies | 1 | 0.3 | Makkah |
| Jouf University | 1 | 0.3 | Al Jouf |
| Qassim University | 1 | 0.3 | Qassim |
| **Total** | **354** | **100.0** |  |

*Note. Institutional labels were based on respondents’ reported affiliations. “Vision University” (n = 21), “Vision College” (n = 1), and “Al-Farabi Private Colleges” (n = 13) were harmonized as Vision Colleges (formerly Al-Farabi Colleges).*

**Supplementary Table S2. Respondents by administrative region of the reported institution (n = 354)**

| **Administrative region** | **n** | **%** |
| --- | --- | --- |
| Riyadh | 300 | 84.7 |
| Makkah | 27 | 7.6 |
| Madinah | 3 | 0.8 |
| Eastern Province | 2 | 0.6 |
| Qassim | 1 | 0.3 |
| Asir | 5 | 1.4 |
| Tabuk | 0 | 0.0 |
| Ha'il | 8 | 2.3 |
| Northern Borders | 0 | 0.0 |
| Jazan | 6 | 1.7 |
| Najran | 0 | 0.0 |
| Al Baha | 1 | 0.3 |
| Al Jouf | 1 | 0.3 |
| **Total** | **354** | **100.0** |

*Note. Regions were assigned from the location of the reported institution. The merged Vision/Al-Farabi affiliation was classified in Riyadh; percentages use the total sample.*
